# Wide-Field Fluorescein Angiography in Wet Age-Related Macular Degeneration

**DOI:** 10.1155/2014/536161

**Published:** 2014-10-14

**Authors:** Savitha Madhusudhan, Nicholas Beare

**Affiliations:** ^1^St Paul's Eye Unit, Royal Liverpool University Hospital, Liverpool L7 8XP, UK; ^2^Department of Eye and Vision Science, Institute of Ageing and Chronic Disease, University of Liverpool, Liverpool L7 8XP, UK

## Abstract

*Purpose*. The aim of our study was to investigate if peripheral retinal ischaemia contributed to the pathogenesis of neovascular AMD (NvAMD), using wide-field fluorescein angiography (WFFA). *Methods*. This prospective study included 30 consecutive patients with newly diagnosed NvAMD in the index eye. Wide-field colour fundus images and fluorescein angiograms were obtained using P200C optomap FA and analysed using a grid with three concentric circles of 50°, 100°, and 200° centred on the fovea to define zones Z1, Z2, and Z3. *Results*. Areas of peripheral retinal nonperfusion were seen in 2 (7%) eyes, peripheral vascular leakage in 5 (17%) eyes, and diffuse dye leakage close to the ora in 5 (17%) eyes. A total of one-third of the study eyes showed changes on WFFA in Z2 and Z3. On comparing index eyes to nonindex eyes in these patients, the presence of NvAMD was associated with peripheral FA changes (P = 0.009, Fisher's test). *Conclusion*. Frank peripheral retinal non-perfusion does not appear to be associated with NvAMD. In some patients with active NvAMD there is degradation of the peripheral blood-retina barrier. Smoking was also found to be associated with the above-mentioned abnormalities.

## 1. Introduction

The pathogenesis of age-related macular degeneration (AMD) is complex with genetic, degenerative, and environmental factors implicated. Hypoxia and ischaemia are thought to play a role in the progression of AMD to neovascular AMD (NvAMD) [1] with attention being previously focussed on the perfusion of the macula. However it is possible that peripheral fundal ischaemia may contribute significantly to an abnormal angiogenic drive, mediated primarily by vascular endothelial growth factor (VEGF). Several vasculopathic factors such as hypertension and smoking, common in the age-group affected by AMD, may be involved in microvascular disease affecting the peripheral retina.

The availability of ultrawide-angle retinal imaging and fluorescein angiography (FA) systems has made it possible to study both morphological and circulatory changes in the retinal periphery. The Reykjavik Eye Study, a population-based epidemiological study, found that eyes with end-stage AMD all had an abnormal peripheral retinal appearance using wide-field colour and autofluorescence imaging [2]. No wide-field fluorescein angiography (WFFA) studies in NvAMD have been published, but Bennett et al. have described hyperfluorescence and leakage in the retinal periphery of patients with NvAMD [3]. Wide-field FA provides an opportunity to study the perfusion of the peripheral fundus of patients with NvAMD.

We aimed to investigate the perfusion of the peripheral retina in NvAMD using WFFA in order to determine if peripheral retinal ischaemia might contribute to NvAMD.

## 2. Materials and Methods

This was a prospective study of patients referred to the AMD Clinic of St. Paul's Eye Unit. Patients were recruited into the study if they were over the age of 50 and were confirmed to have NvAMD from initial clinician examination and baseline imaging which included conventional fluorescein and indocyanine green angiography at the point of referral. Exclusion criteria were polypoidal choroidal vasculopathy, presence of other ocular diseases expected to affect the peripheral retina, any preproliferative or proliferative diabetic retinopathy, previous peripheral retinal argon laser, previous treatment with intravitreal steroids, or anti-VEGF drugs and history of myopia of more than eight dioptres. Thirty consecutive patients were included and the eye with the presenting symptoms was designated as the index eye.

Permission to investigate the use of WFFA instead of pretreatment repeat conventional FA was obtained from the hospital Audit and Information Department as part of service evaluation of WFFA in AMD.

As per the departmental protocol, a complete ocular and systemic history and best corrected visual acuity (BCVA) in both index and fellow eyes were recorded. OCT imaging (Spectralis) of the macula in both eyes was performed. After obtaining informed consent from the patients the P200C Optomap FA (Optos Plc, Dunfermline, Scotland, UK) ultrawide-field imaging system was used to obtain colour fundus photographs in both eyes and a fluorescein angiogram. A protocol was developed for WFFA with the ophthalmic imaging team to perform the early run of the angiogram on the index eye and also optimise superior and inferior retinal views.

**Figure 1:**
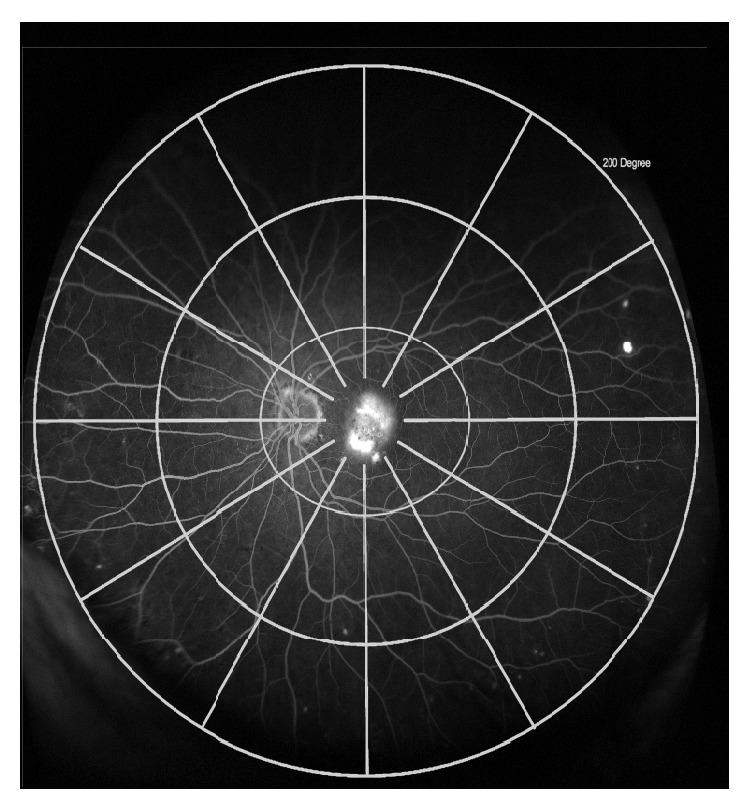
Grid superimposed on wide-field fluorescein angiogram image to show zones 1 (<50°), 2 (50–100°), and 3 (100–200°) centred on the fovea.

Optomap fundal images were analysed and graded by the authors. We superimposed a grid of 3 concentric circles of 50, 100, and 200 degrees centred on the fovea to define zones Z1 (<50°), Z2 (50–100°), and Z3 (100–200°), respectively; each was divided into 12 hours (Figure 1). The primary objective was to detect the presence of peripheral perfusion abnormalities and define the extent of involvement. The secondary objectives were to correlate the above changes with the subtype of NvAMD lesion, prevalence of systemic vascular disease and smoking, and to describe any other peripheral retinal changes normally associated with AMD.

Fisher's exact test was used to correlate the presence of peripheral FA changes with NvAMD in the index eyes and with hypertension and smoking in independent subgroup analyses.

## 3. Results

The study included 30 Caucasian patients, 19 female and 11 male, with an average age of 78. There were 14 right eyes and 16 left eyes; 6 of the 30 eyes were pseudophakic and the rest were phakic; 3 eyes were on topical prostaglandin analogues for either glaucoma or ocular hypertension; 1 eye had received PDT for NvAMD seven years previously.

Cardiovascular or circulatory disease included hypertension in 20 (67%) patients, myocardial infarction in 6 (20%) patients, stroke in 5 (17%) patients, and diabetes mellitus in 2 patients (7%). Nineteen (63%) patients had a history of smoking, either previous or current. Seven (23%) patients were on oral antioxidant and vitamin supplements recommended for AMD.

The mean BCVA was 53 ETDRS letters. The NvAMD lesion subtype in these 30 eyes was categorised as follows: wholly classic choroidal neovascularisation (CNV) in 7 (23%), occult fibrovascular pigment epithelial detachment (FvPED) in 11 (37%), minimally classic CNV in 5 (17%), predominantly classic CNV in 2 (7%), and retinal angiomatous proliferation (RAP) in 5 (17%).

### 3.1. Wide-Field Colour Image Findings

**Figure 2:**
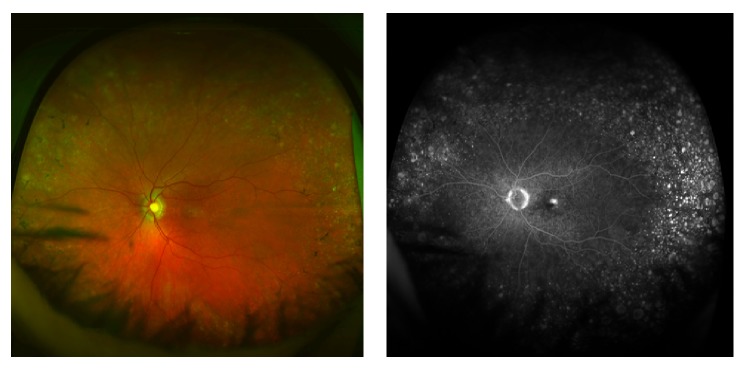
Optomap colour and fluorescein angiogram images showing extensive drusen, RPE mottling, and small areas of RPE atrophy in the retinal periphery in an eye with retinal angiomatous proliferation.

Drusen were seen in Z2 in 18 (60%) eyes and in Z3 in 20 (67%) eyes on wide-field colour photos (Figure 2), ranging from a few scattered drusen to 12 clock hours distribution. Drusen were seen in Z2 and Z3 in a total of 83% of eyes and in a similar proportion of eyes in patients with hypertension and history of smoking (80% and 79%, resp.). Mottling of the RPE was seen in 3 (10%) eyes in Z2 and 10 (33%) eyes in Z3. Isolated areas of RPE atrophy were seen in 3 (10%) eyes in Z2 and 4 (13%) eyes in Z3. These changes did not show any relationship to either the NvAMD lesion subtype or visual acuity level.

### 3.2. Wide-Field Fluorescein Angiography Findings

**Figure 3:**
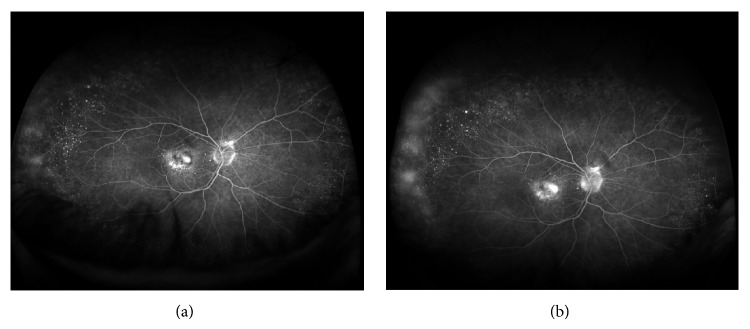
Wide-field fluorescein angiogram images at one (a) and five (b) minutes of the run showing vascular leakage in the temporal retinal periphery in an eye with minimally classic choroidal neovascularisation.

**Figure 4:**
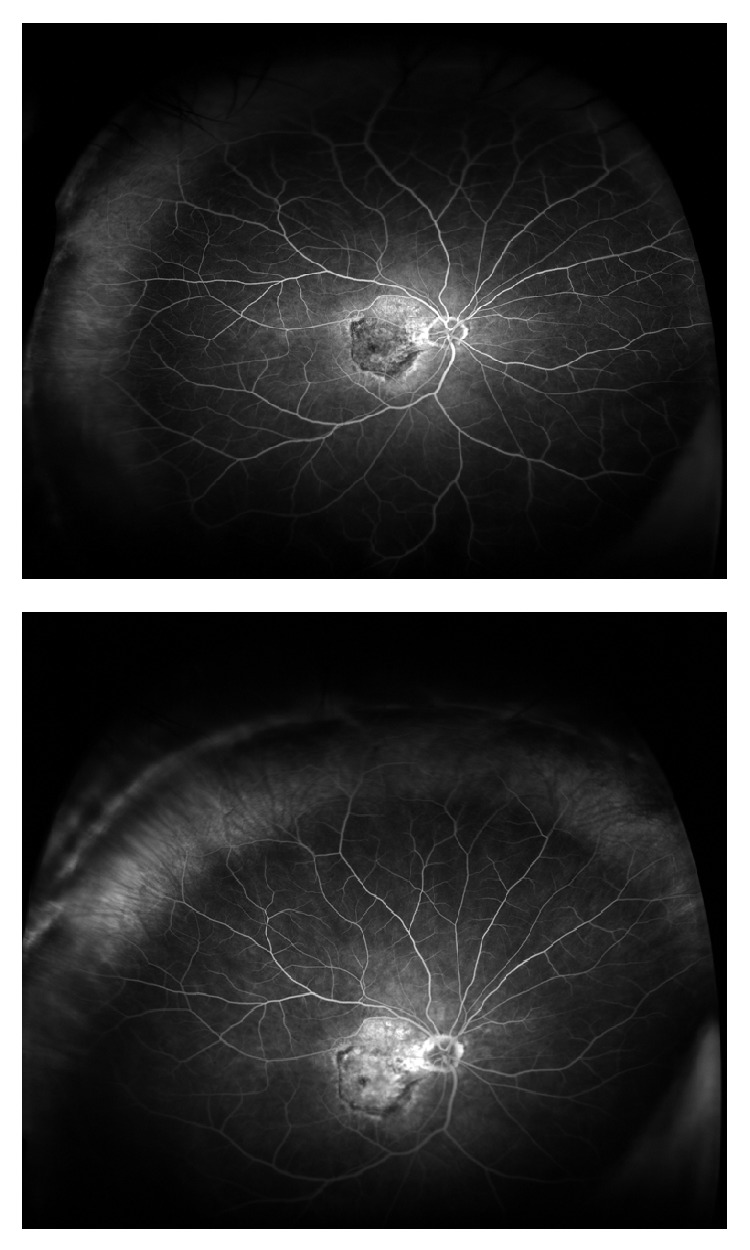
Diffuse leakage and hyperfluorescence in zone 3 (100–200°) in an eye with active fibrovascular pigment epithelial detachment.

The peripheral WFFA findings are presented in Table 1. Areas of peripheral retinal nonperfusion were seen in 2 eyes. This was a small area (1/3 of a disc area) in one in Z3. In the second, there were 4 hours of nonperfusion in Z3, associated with collateral vessel formation, with no changes in Z1 and Z2. There was leakage from peripheral retinal vessels (Z3) without associated nonperfusion in 5 (17%) eyes (Figure 3). These were 1 to 4 hours in extent. Indeterminate hyperfluorescence and diffuse dye leakage were seen in 5 (17%) eyes mostly involving or close to the ora serrata (Figure 4). WFFA images were not interpretable in 1 eye due to poor quality.

### 3.3. Systemic Associations of WFFA Abnormalities

A total of 10 (33%) eyes had one or more of the above three WFFA features in the study eye (nonperfusion, vascular leakage, or diffuse leakage). Of these 10 patients, hypertension was present in 9, history of cerebrovascular accident in 4, myocardial infarction in 3, and angina and diabetes in 1 each.

Peripheral WFFA changes were present in 10 (53%) of 19 patients with a history of smoking but not in any of the 11 nonsmokers (0%), which was significant (*P* = 0.0040, Fisher's test) (Table 2). Peripheral WFFA changes were present in 9 (45%) out of the 20 patients with hypertension compared to 1 (10%) out of the 10 patients with no history of hypertension. This difference was not statistically significant (*P* = 0.1008). 16 (53%) patients had a history of both hypertension and smoking, and 9 (56%) of them had peripheral WFFA changes (*P* = 0.0067).

WFFA changes were present in 3 (50%) of those with a history of myocardial infarction, 4 (80%) with a history of stroke, and 1 (50%) with diabetes, all of whom had a history of smoking, including 2 current smokers. WFFA changes were present in 1 (14%) of the patients on AMD-specific vitamin supplements.

### 3.4. Comparison with Non-Index Eyes

With regard to non-index eyes, 15 had dry AMD, 9 had inactive NvAMD (macular fibrosis), and 6 eyes had a normal macula. 5 patients had peripheral dye leakage, either vascular or diffuse in the non-index eye; all these eyes had early dry AMD or a normal macula (Table 1). Two of these 5 patients only had peripheral WWFA changes in the non-index eye (both smokers).

Out of the 10 patients with peripheral vascular abnormalities in the index eye, we compared index to non-index eyes in 9 patients (1 patient with artificial eye excluded). Given that all systemic factors were matched, we found that the presence of active NvAMD was associated with peripheral FA changes (100% versus 33%, *P* = 0.009, Fisher's test) (Table 3).

## 4. Discussion

The use of wide-field fundus fluorescein angiography was first reported in 2005 as a valid means of evaluating peripheral retinal perfusion [4]. Wide-field imaging is proving useful in diagnosing and managing a wide range of vitreoretinal conditions including diabetic retinopathy [5], vascular occlusions, posterior uveitis [6], and peripheral fundal tumours. The P200C Optomap FA scanning laser ophthalmoscope system captures a high resolution (3000 × 3000 pixels) 200 degree view of the retina in the horizontal meridian. The width of the image field in the vertical meridian is not as reliably wide due to impingement of lashes and lids. Despite these artefacts, it still provides a much wider field of view than is possible with conventional FA.

The Alienor study, a population-based epidemiological study of people over 75, found 52.8% prevalence of features associated with AMD including drusen, RPE changes, and atrophy in the peripheral retina using Optomap imaging [7]. Although the number of eyes with NvAMD in this series was small, they report that all eyes with end-stage AMD had abnormal retinal periphery findings on colour and autofluorescence images. In our cohort of patients with NvAMD, these peripheral changes occurred in 97% implying that the prevalence of these features, associated with an aging retina, is ubiquitous in eyes with active NvAMD. Wide-field retinal imaging using Optomap has also helped to show that abnormalities in peripheral fundus autofluorescence (FAF) are more prevalent in AMD, compared to normal controls [8].

The vulnerability of the macula to choroidal neovascularisation is considered to be due to certain features including high oxygen tension and oxidative stress, high photoreceptor density leading to more lipofuscin formation, increased exposure to light, and a thinner Bruch's membrane. In addition there are studies showing evidence of reduced choroidal and retinal blood flow in AMD [9, 10]. We sought to investigate whether abnormal perfusion in the peripheral retina may be a significant factor in the pathogenesis of NvAMD in a typical AMD cohort in the UK with an expected level of cardiovascular comorbidity. This was considering that, in conditions like diabetic retinopathy, untreated peripheral nonperfusion and late peripheral vascular leakage on WFFA are associated with retinal neovascularisation [11].

In our series only 2 patients showed areas of retinal nonperfusion. One was very small and one was thought to be related to a peripheral branch retinal vein occlusion. It therefore seems unlikely that demonstrable retinal nonperfusion on WFFA is a factor in the development of NvAMD.

Thirty percent of our patients had peripheral vascular leakage and diffuse hyperfluorescence, which ranged between one to four hours mostly affecting the temporal retina. There was 360° hyperfluorescence at the ora in 2 eyes. It is not clear whether this leakage of fluorescein adjacent to the ora is physiological or abnormal. However, it is possible that the leakiness of the blood-retina barrier adjacent to the ora and in peripheral zones is indicative of chronic hypoxia without frank nonperfusion. Retinal capillary density is the lowest at the far periphery; therefore, these changes are probably most pronounced at the very periphery of the neurosensory retina. While dye leakage from peripheral vessels is suggestive of a breakdown of the inner blood-retinal barrier, diffuse nonspecific dye leakage and hyperfluorescence may be, in addition, secondary to a breakdown of the outer blood-retinal barrier or loss of RPE integrity. Bennett et al. have described similar peripheral hyperfluorescence and leakage in a higher proportion of eyes with wet AMD than in our series [3]. This difference could be due to differences in patient profile, systemic factors, and stage of NvAMD.

It will be useful to explore the chronological relationship between peripheral changes and the onset and evolution of macular disease through long-term prospective studies combining ultrawide-field autofluorescence and FA. If peripheral perfusion abnormalities precede central fundal changes, and are picked up early, this may provide an opportunity for preventative treatment to preserve central vision.

We recognise that the shortcomings of our study are the small number of recruits and the lack of a control group. With the exception of a peripheral branch retinal vein occlusion in one patient the absence of significant peripheral ischaemia in 30 patients gives a clear indication that it is unlikely to be a factor in NvAMD in this population. However a control group would have been informative in assessing the significance of the peripheral leakage found. We were able to compare eyes with active NvAMD matched to eyes without it, in the same patient.

In conclusion, although our study using WFFA did not find significant retinal nonperfusion in patients with NvAMD, making it unlikely that nonperfusion is a factor in choroidal neovascularisation, peripheral leakage, due to degradation of the blood-retina barrier, was seen in a proportion of patients with NvAMD. Interestingly, when index eyes with WFFA abnormalities were compared to fellow eyes without active NvAMD, peripheral FA changes were associated with active NvAMD. Likewise the strong association between smoking and peripheral fluorescein leakage suggests an effect on blood-retina barrier integrity. While the reason why some patients and not others develop new vessels in the choroid and retina is still unknown, this study adds to our knowledge and indicates that further studies of the very peripheral fundus are warranted in order to improve our understanding of the pathogenesis of wet AMD.

## Figures and Tables

**Table 1:** Neovascular AMD lesion subtype, best-corrected visual acuity (BCVA), and wide-field fluorescein angiography findings in index eyes; macular status in fellow eyes.

Study eye						Non-study eye		
Patient	NvAMD lesion subtype	BCVA (ETDRS letters)	Nonperfusion on WFFA	Leakage from blood vessels on WFFA	Indeterminate hyperfluorescence on WFFA	Macular status	BCVA (ETDRS letters)	WFFA findings
1	Classic CNV	26			Z3: diffuse leakage temporally	Normal	86	Z3: leakage from 3 vessel segments over 1 hr, temporal

2	Classic CNV	38				Inactive wet AMD	10	

3	Classic CNV	32				Early dry AMD	67	Z3: leakage over 4 hours, temporal; adjacent nonperfused area

4	Classic CNV	50			Z2 and Z3: drusen-staining and late leakage	Inactive wet AMD	1	

5	Classic CNV	34		Z3: 1 hour; superotemporal		Advanced dry AMD	34	

6	Classic CNV	43				Early dry AMD	63	

7	Classic CNV	51				Inactive wet AMD	23	

8	Occult FVPED	23			Z3: complete ring of leakage at ora	Early dry AMD	85	

9	Occult FVPED	62				Early dry AMD	85	

10	Occult FVPED	18	Z3: 1/3rd disc area			Artificial eye	—	

11	Occult FVPED	65		Z3: 3 hours; temporal	Z3: complete ring of leakage at ora	Advanced dry AMD	39	

12	Occult FVPED	50				Early dry AMD	89	

13	Occult FVPED	40				Early dry AMD	81	

14	Occult FVPED	62	Poor quality images			Inactive wet AMD	13	

15	Occult FVPED	55				Early dry AMD	77	Z3: leakage from 2 small capillaries, temporal

16	Occult FVPED	77			Z3: leakage at temporal ora	Early dry AMD	90	

17	Occult FVPED	78				Normal	85	

18	Inactive FVPED	54		Z3-2 hours; temporal		Early dry AMD	79	

19	Minimally classic	74	Z3: 4 hours; temporal	Z3: 4 hours; temporal; collaterals	Z2 and Z3: drusen-staining and late leakage	Early dry AMD	86	Z3: diffuse leakage temporally

20	Minimally classic	59				Normal	84	

21	Minimally classic	44				Early dry AMD	74	

22	Minimally classic	48				Early dry AMD	10	

23	Minimally classic	69		Z3: 1 hour; temporal		Inactive wet AMD	7	

24	Predominantly classic with FVPED	60				Inactive wet AMD	78	Z3: pronounced RPE window defect, temporal

25	Predominantly classic with FVPED	29				Early dry AMD	76	

26	RAP	68				Inactive wet AMD	31	

27	RAP	80				Early dry AMD	75	

28	RAP	72				Early dry AMD	92	

29	RAP	61			Z2 and Z3: drusen-staining and late leakage	Normal	85	

30	Inactive RAP	60			Z3: diffuse leakage temporally	Inactive wet AMD	60	Z3: diffuse leakage temporally

**Table 2:** Number of index eyes showing the presence of peripheral retinal perfusion abnormalities in subgroups of patients with a history of smoking and hypertension.

Peripheral perfusion abnormalities	Smoking		Hypertension	
	Yes	No	Yes	No
Present	10	0	9	1
Absent	9	11	11	9

**Table 3:** Number of fellow eyes in patients with peripheral perfusion abnormalities in the index eyes showing similar changes.

Peripheral perfusion abnormalities	Index eye	Fellow eye
Present	9	3
Absent	0	6
